# Evaluation of clinical performance of artificial intelligence-assisted measurement of thoracic aortic diameter on non-contrast CT

**DOI:** 10.1097/MD.0000000000050702

**Published:** 2026-09-18

**Authors:** Akiyoshi Suzuki, Nobuo Tomizawa, Kotaro Yamamoto, Yuichi Morita, Shohei Fujita, Katsuhiro Sano, Toshiaki Akashi, Mitsuo Nishizawa, Kazuhiro Suzuki, Akihiko Wada, Daisuke Endo, Satoshi Matsushita, Kanako K. Kumamaru, Keiichi Funaki, Jonathan Sperl, Minoru Tabata, Shigeki Aoki

**Affiliations:** a Department of Radiology, Juntendo University Graduate School of Medicine, Tokyo, Japan; b Department of Radiology, Kokura Memorial Hospital, Kitakyushu, Japan; c Department of Radiology, The University of Tokyo, Tokyo, Japan; d Athinoula A. Martinos Center for Biomedical Imaging, Massachusetts General Hospital, Boston, MA; e Department of Cardiovascular Surgery, Juntendo University Graduate School of Medicine, Tokyo, Japan; f Digital & Automation Department, Siemens Healthcare K.K., Tokyo, Japan; g Diagnostic Imaging, Computed Tomography, Clinical Concepts, Siemens Healthineers AG, Forchheim, Germany.

**Keywords:** aortic aneurysm, artificial intelligence, multidetector computed tomography, thorax

## Abstract

This study aimed to evaluate the clinical performance of artificial intelligence (AI)-assisted measurement of thoracic aortic diameter on non-contrast computed tomography (NCCT). This single-center prospective study included 79 patients (mean age = 70 ± 13 standard deviation; 46 men) who underwent NCCT and arterial phase contrast-enhanced computed tomography (CECT) for evaluating aortic diseases between 2021 and 2022. Maximum diameters of 9 American Heart Association landmarks and the maximum ascending/descending aorta were measured. Upper limits of the 95% confidence interval (CI) for the absolute difference in aortic diameter between the AI-assisted/manual method on NCCT and the manual method on CECT (Diff_AI-assisted_/Diff_manual_) were calculated and compared using a predefined cutoff of 1.5 mm. Evaluation times were also compared. No significant difference was found between per-patient Diff_AI-assisted_ and Diff_manual_ (1.10 mm; 95% CI = 1.03–1.18 vs 1.03 mm; 95% CI = 0.95–1.11, *P* = .058); the upper limit of the 95% CI was <1.5 mm. The upper limit of the 95% CI of the geometric mean Diff_AI-assisted_ and Diff_manual_ was also <1.5 mm at each landmark. The median evaluation time of the AI-assisted method was shorter than that of the manual method (expert radiologist = 272 seconds [interquartile range (IQR) = 229–313] vs 496 seconds [IQR = 438–550]; *P* < .001, training radiologist: 304 seconds [IQR = 257–404] vs 710 seconds [IQR = 563–855]; *P* < .001). AI-assisted thoracic aortic diameter measurement on NCCT was as accurate as manual measurement on CECT; its evaluation time was shorter than that of manual measurement on NCCT.

## Graphical abstract

**Figure:**
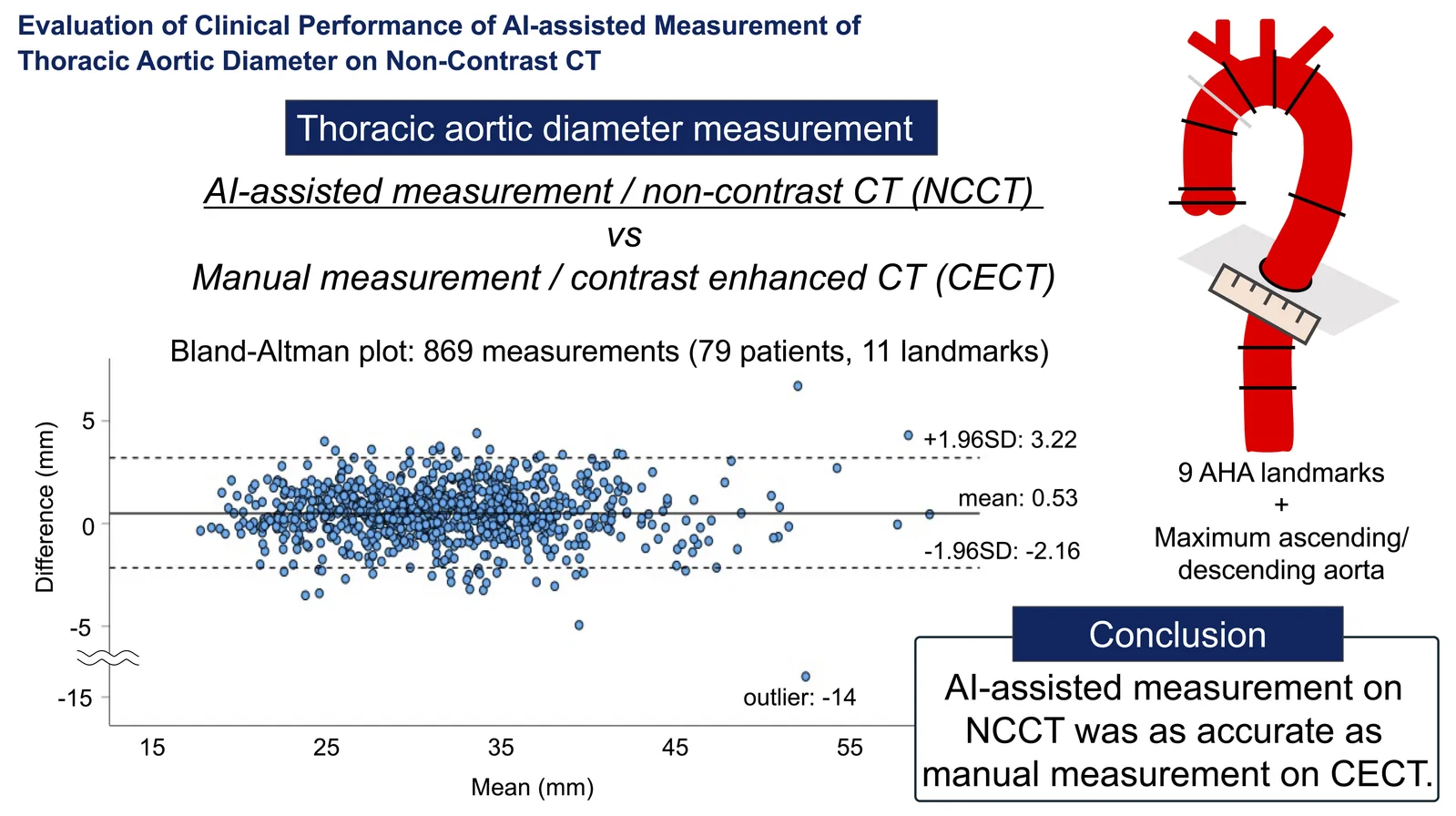

## 1. Introduction

Thoracic aortic aneurysm is a common manifestation of aortic disease worldwide. Its prevalence is increasing, with an estimated 5 to 10 out of 100,000 individuals being diagnosed with aortic aneurysms per year.^[1–4]^ Thoracic aortic aneurysms are usually asymptomatic and identified incidentally on imaging examinations, such as chest radiography. However, these aneurysms can cause fatal bleeding if rupture occurs. Accurate measurement of aortic diameter is essential for guideline-directed management. According to the American Heart Association and European Society of Cardiology guidelines for thoracic aortic diseases, the treatment procedure for thoracic aortic aneurysm is decided based on aneurysm size, growth rate, location, and coexisting connective tissue disease (such as Marfan syndrome). For instance, asymptomatic patients with aneurysms of the aortic root or ascending aorta exceeding 55 mm in diameter should be considered for surgical repair.^[2,5]^

Computed tomography (CT) is widely used as the first choice for evaluating aortic diameter. Arterial phase contrast-enhanced CT (CECT) can delineate the entire aorta and its branches in a short breath-holding time, allowing for distinction between the wall and lumen. Moreover, using the obtained thin-slice data, 3-dimensional images can be reconstructed, and cross-sections perpendicular to the centerline of the aorta can be obtained.^[1,6]^ Additionally, using electrocardiogram (ECG)-gated images decreases motion artifact and improves edge depiction during aortic root imaging, reducing measurement variability.^[7]^ However, contrast media is associated with potential risks, including allergic reaction and contrast-induced kidney injury. Furthermore, ECG-gated scans are time-consuming and complicated. Therefore, they are not suitable for screening, routine follow-up, or in patients with a history of allergic reaction to contrast media or chronic kidney disease.

Recent advancements in artificial intelligence (AI) have increased its potential application in medicine. Several studies have shown that CECT AI-only measurement of aortic diameter is as accurate as that of manual measurement. A recently developed AI-based prototype includes post-processing software for chest CT images that could provide an automatic measurement of thoracic aortic diameter on both CECT and non-contrast CT (NCCT). NCCT is easy to perform and does not require contrast media, which could lead to adverse reactions. However, variations have been reported in the results of AI-only measurement on NCCT.^[8]^ We hypothesized that the variations could be reduced by AI-assisted measurement, resulting in more effective and accurate assessment.

Therefore, this study aimed to evaluate the clinical performance of AI-assisted measurement of thoracic aortic diameter on NCCT.

## 2. Materials and methods

### 2.1. Patients

This single-center prospective study was approved by the Institutional Review Board of Juntendo University Hospital, Tokyo, Japan (E21-0170), and written informed consent was obtained from all patients. Initially, 93 patients were included between December 2021 and December 2022. The inclusion criteria were patients without a history of open or endovascular thoracic aortic surgery and those who were clinically indicated for non-ECG-gated NCCT and arterial phase CECT for evaluating aortic disease. The exclusion criteria were unavailable consent (n = 6), poor kidney function (estimated glomerular filtration rate <30 mL/min/1.73 m^2^, n = 5), history of previous adverse reaction to iodinated contrast media (n = 2), and unavailable AI software analysis (n = 1).

### 2.2. CT protocol

A 320-row CT scanner was used for imaging the aorta (Aquilion ONE Prism Edition, Canon Medical Systems). Non-ECG-gated NCCT and arterial phase CECT were performed using the following parameters: tube voltage, 120 kVp; tube current, determined by automated exposure control (Volume EC, Canon Medical Systems, target standard deviation [SD] = 12); detector width, 80 × 0.5 mm; rotation time, 0.5 s/rotation; and pitch factor, 0.813. Regarding CECT, the patients were administered 18.0 mgI/kg/s of iodine as iopamidol (Iopamiron 370, Bayer) until attenuation in the descending aorta reached the predefined threshold, followed by a 30 mL saline flush. The bolus-tracking method was used to determine the scan timing. The scan began 6 seconds after the enhancement of the descending aorta had reached 150 HU.^[9]^ The images were reconstructed with a section thickness of 1 mm and an increment of 1 mm using an FC13 convolution kernel with deep learning reconstruction (Advanced Intelligent Clear-IQ Engine, body sharp, mild reconstruction strength, Canon Medical Systems). The images were transferred to post-processing workstations (syngo.via VB60A, Siemens Healthineers, and Synapse Vincent version 6.0, Fujifilm Medical).

### 2.3. Post-processing of images

The AI-Rad Companion Research Chest CT Explore prototype (not for clinical use; Siemens Healthineers) is a post-processing application for contrast-enhanced and non-contrast-enhanced chest CT images. It automatically provides the cross-section and maximum aortic diameter at 9 anatomical landmarks according to the American Heart Association guidelines. The landmarks included the following: landmark 1, the sinuses of Valsalva; landmark 2, the sinotubular junction; landmark 3, the mid-ascending aorta; landmark 4, the proximal aortic arch (aorta at the origin of the brachiocephalic trunk); landmark 5, the mid-aortic arch (between the left common carotid and subclavian arteries); landmark 6, the proximal descending thoracic aorta (approximately 2 cm distal to the left subclavian artery); landmark 7, the mid-descending aorta (level of the pulmonary arteries as easily identifiable landmarks); landmark 8, at the diaphragm; landmark 9, at the celiac artery origin; additionally, at the maximum diameters of the ascending and descending aorta, respectively (landmarks 10 and 11). The obtained NCCT images were then transferred to syngo.via, after which AI analysis was performed automatically. The original images and results of the analysis can be reviewed on syngo.via. The AI algorithm first detects and segments the aorta using a deep-learning-based segmentation architecture.^[10]^ Based on the aortic centerline and the outer segmentation mask, orthogonal reformats perpendicular to the centerline are generated, from which the aortic diameters are determined.

### 2.4. Aortic diameter measurement

**Figure 1.**
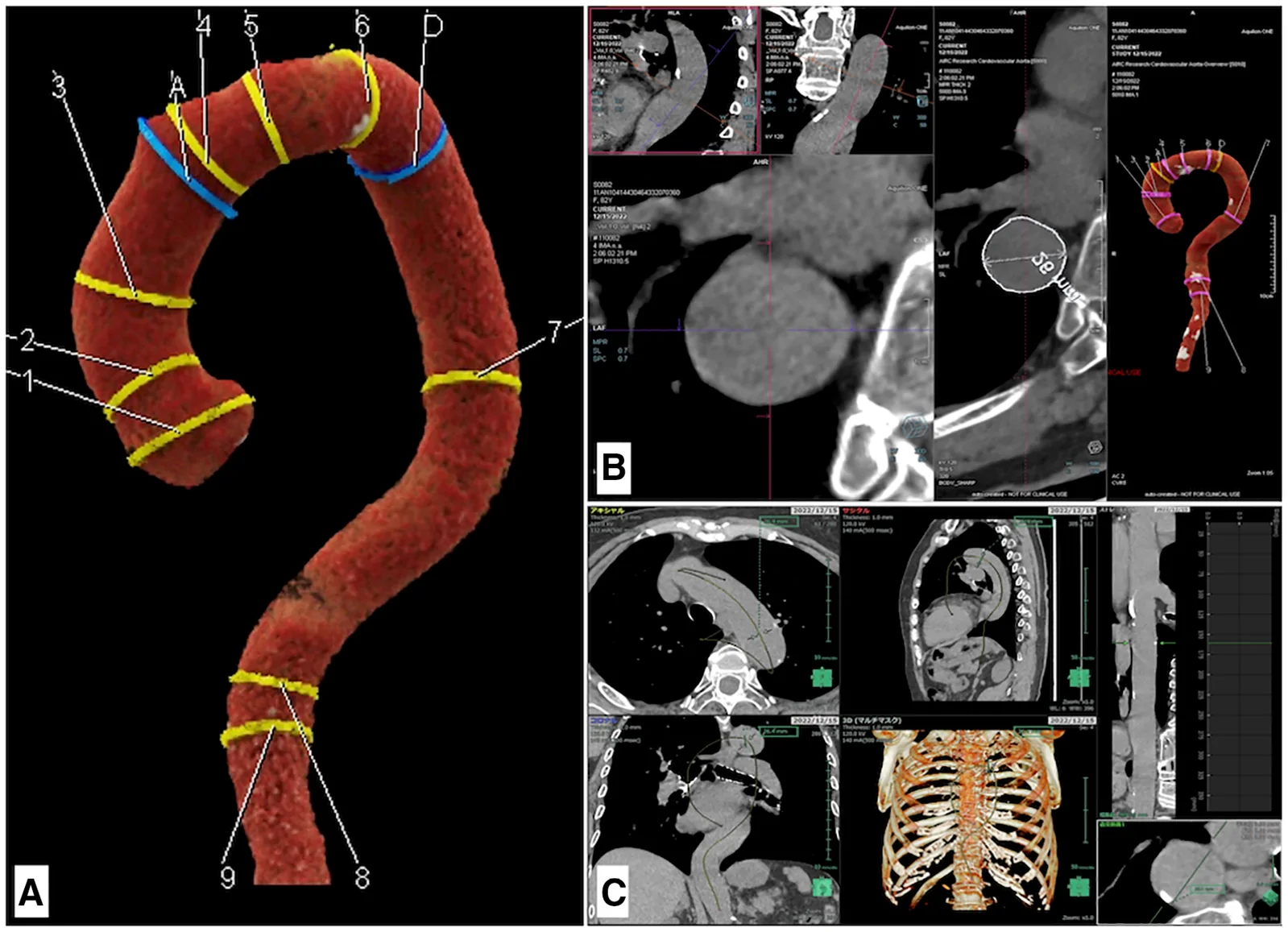
The measured landmarks of thoracic aortas and the actual measurement interface. The thoracic aorta and its standard anatomical landmarks for measuring the aortic diameter according to the American Heart Association (yellow lines 1–9), including the largest diameter in the ascending and descending aorta (blue lines: A, largest diameter of the ascending aorta; D, largest diameter of the descending aorta), are graphically illustrated by AI (A). The actual interface of the AI-assisted measurement is shown. The selected landmark and the diameter measured by AI in the corresponding cross-section were arranged to be shown together (B). The actual interface of the manual measurement is shown. Manual curved planar reformation and processing were performed by the viewer (C). AI = artificial intelligence.

Two independent radiologists (radiologist 1, a cardiovascular imaging specialist; radiologist 2, a radiology resident who did not specialize in cardiovascular imaging, with 15 and 4 years of experience, respectively) measured the maximum cross-sectional diameter of the thoracic aorta at the 11 landmarks. The diameter was defined as the length of the largest external diameter in the measurement plane perpendicular to the aortic centerline. The aortic root was measured using the sinus-to-commissure method.^[11]^ The measurements were performed for the following 3 datasets: NCCT by the AI-assisted method, NCCT by the manual method, and CECT by the manual method. Regarding the AI-assisted measurement, the radiologists determined whether the AI-presented sections and measured diameters were correct. Then, they manually remeasured the maximum diameters using the AI output as a reference without adopting the existing AI results. Considering the manual method on NCCT, straightened curved planar reformation (CPR) was achieved by manually plotting the center points of the aortic sections and then reconstructing the images based on the centerline on the workstation. After making manual fine adjustments, the aortic diameters were measured (Fig. 1). The measurements on CECT were used as ground truth because, with the use of contrast media, straightened CPR could be obtained automatically and more accurately on the workstation. In addition, the boundary between the aortic wall and surrounding structures, such as atelectasis, could be clarified.

The absolute difference in aortic diameter between the AI-assisted method on NCCT and the manual method on CECT (Diff_AI-assisted_) and that in aortic diameter between the manual method on NCCT and the manual method on CECT (Diff_manual_) were calculated for each landmark. The mean Diff_AI-assisted_ and Diff_manual_ for the 11 landmarks were defined as per-patient Diff_AI-assisted_ and Diff_manual_, respectively. To avoid recall bias, each reading was performed after a 3-week washout period. The mean diameters measured by the 2 radiologists at each landmark were used for further analyses.

Evaluation times for the 11 landmarks were also recorded. We defined the evaluation time as the sum of the manual CPR processing time and measurement time. Manual CPR processing time was defined as the time required to create a straightened CPR image to acquire a cross-sectional image. AI processing was performed immediately after the CT scan; the results were available at the time of the reading session. Therefore, we determined the manual CPR processing time for AI-assisted measurement to be 0 seconds.

### 2.5. Statistical analysis

Statistical analyses were performed using a commercially available statistical package (JMP Pro version 16.0.0; SAS Institute Inc.). Quantitative variables are expressed as means ± SDs. Categorical variables and basic characteristic variables are expressed as numbers or percentages, unless otherwise described. The inter-rater reliability between the 2 radiologists was evaluated using intraclass correlation coefficients in SPSS Statistics version 29.0.0.0 (IBM). Since the per-landmark Diff_AI-assisted_ and Diff_manual_ had right-skewed distributions, they were first calculated on a logarithm-transformed scale (log [data + 1]) and then transformed back. The addition of 1 to the variables before the logarithmic transformation allowed us to include all patients and landmarks with differences of 0 between the measured aortic diameter and ground truth using the analyses.

Based on previous studies reporting measurement variability in aortic diameter assessment, as well as current clinical guidelines recognizing that differences of approximately 1 to 2 mm are difficult to assess consistently due to measurement error and interobserver variability, a difference of <1.5 mm between the measured aortic diameter and the reference standard was considered unlikely to represent a clinically meaningful discrepancy in this study.^[8,11–14]^ Furthermore, this threshold was considered clinically relevant because management decisions in thoracic aortic disease are based on substantially larger changes in aortic diameter, such as rapid aortic enlargement (e.g., ≥5 mm within 6 months), rather than differences of only 1 to 2 mm.^[1,2]^ We adopted this approach. The upper limits of the 95% confidence interval (CI) of Diff_AI-assisted_ were calculated and compared with the predefined cutoff of 1.5 mm. These results were used as the primary endpoints. The upper limits of the 95% CI of Diff_manual_ were also calculated and compared with the cutoff and the Diff_AI-assisted_. To detect an effect size of dz = 0.375 (mean of difference, 1.5; SD of difference, 4) with 80% power (alpha = .05, two-tailed), a priori power analysis suggested that 58 patients would be needed in a paired *t* test. Considering that some patients were excluded, the target number of patients was set at 80. A *P* value of < .05 was considered statistically significant.

## 3. Results

### 3.1. Patient characteristics

**Figure 2.**
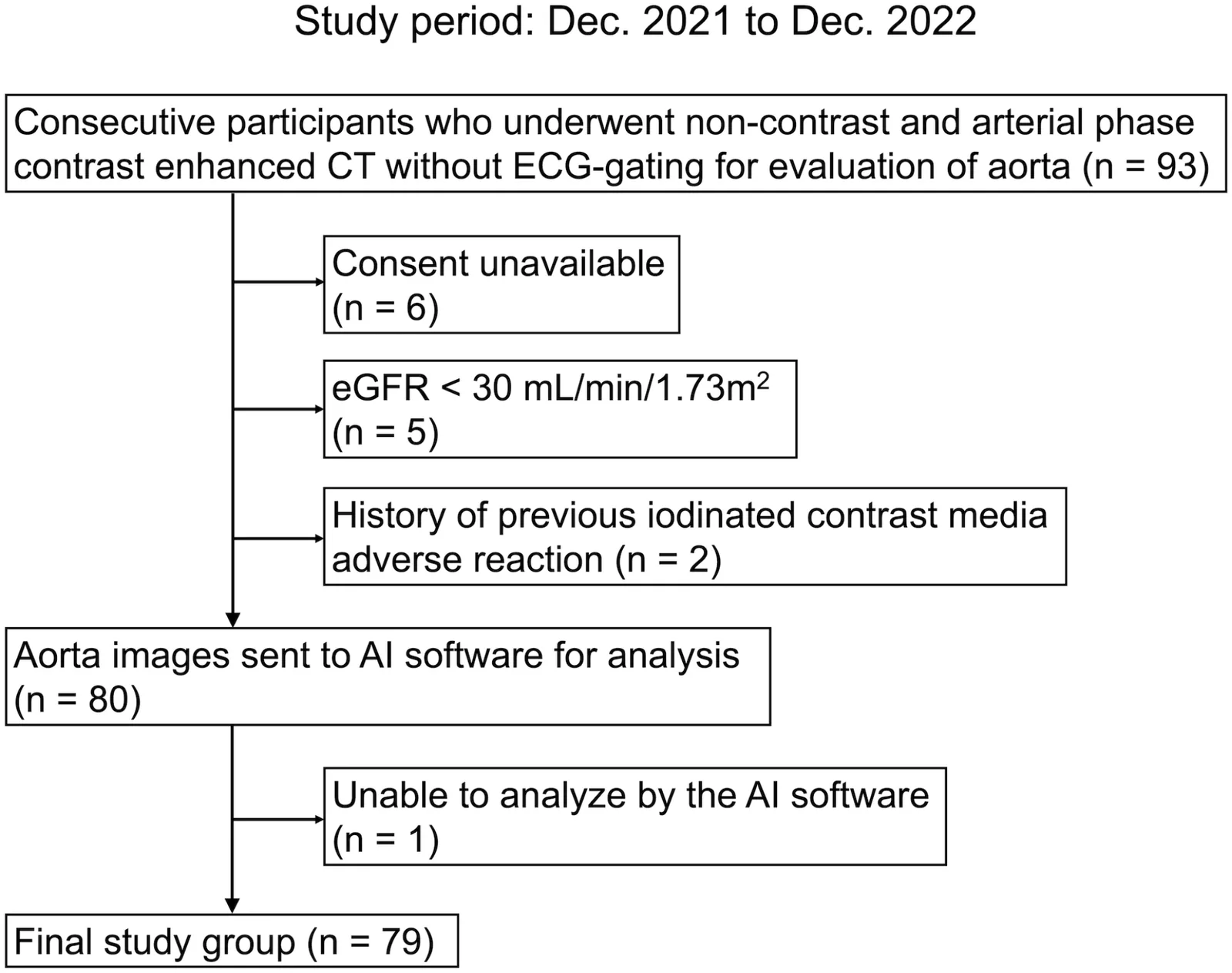
Patient selection flowchart. AI = artificial intelligence, CT = computed tomography, ECG = electrocardiogram, eGFR = estimated glomerular filtration rate.

A total of 79 patients (mean age = 70 years ± 13 [SD]; 46 men) were finally enrolled in this study. The patient selection flowchart is shown in Figure 2. The clinical characteristics and contrast administration details are summarized in Table 1. No adverse effects were observed in any of the patients.

**Table 1:** Patient demographics.

Number of patients	79
Male/female	46/33 (58/42)
Age (y)	70 ± 13
Body mass index (kg/m^2^)	23.5 ± 4.0
Contrast dose (mL)	68.4 ± 14.0
Injection duration (s)	22.8 ± 3.8
Indication	
Abdominal aortic aneurysm	32 (41)
Thoracic aortic aneurysm	27 (34)
Dissection of the aorta	16 (20)
Iliac artery aneurysm	2 (3)
Renal artery aneurysm	1 (1)
Superior mesenteric artery dissection	1 (1)

Unless otherwise noted, values are either the number of patients with percentages in parentheses or mean ± standard deviation.

### 3.2. Per-patient diameter measurement analysis

The mean per-patient aortic diameters measured by the AI-assisted and manual methods, and per-patient ground truth were 32.0 mm ± 3.9, 31.7 mm ± 3.9, and 31.5 mm ± 3.9, respectively. No difference was found between the mean per-patient Diff_AI-assisted_ and Diff_manual_ (1.10 mm; 95% CI = 1.03–1.18 vs 1.03 mm; 95% CI = 0.95–1.11; *P* = .058). Both upper limits of the 95% CI were less than the cutoff of 1.5 mm. The mean per-patient Diff_AI-assisted_ and Diff_manual_ according to the indication disease were as follows: abdominal aortic aneurysm (n = 32), 1.02 mm; 95% CI = 0.92 to 1.12 versus 0.94 mm; 95% CI = 0.85 to 1.02; *P* = .14; thoracic aortic aneurysm (n = 27), 1.13 mm; 95% CI = 0.96 to 1.30 versus 1.14 mm; 95% CI = 0.95 to 1.32; *P* = .97; aortic dissection (n = 16), 1.28 mm; 95% CI = 1.12 to 1.46 versus 1.01 mm; 95% CI = 0.85 to 1.17; *P* = .004; and other diseases (n = 4), 1.00 mm; 95% CI = 0.51 to 1.49 versus 1.17 mm; 95% CI = 0.65 to 1.68; *P* = .34.

### 3.3. Per-landmark diameter measurement analysis

**Figure 3.**
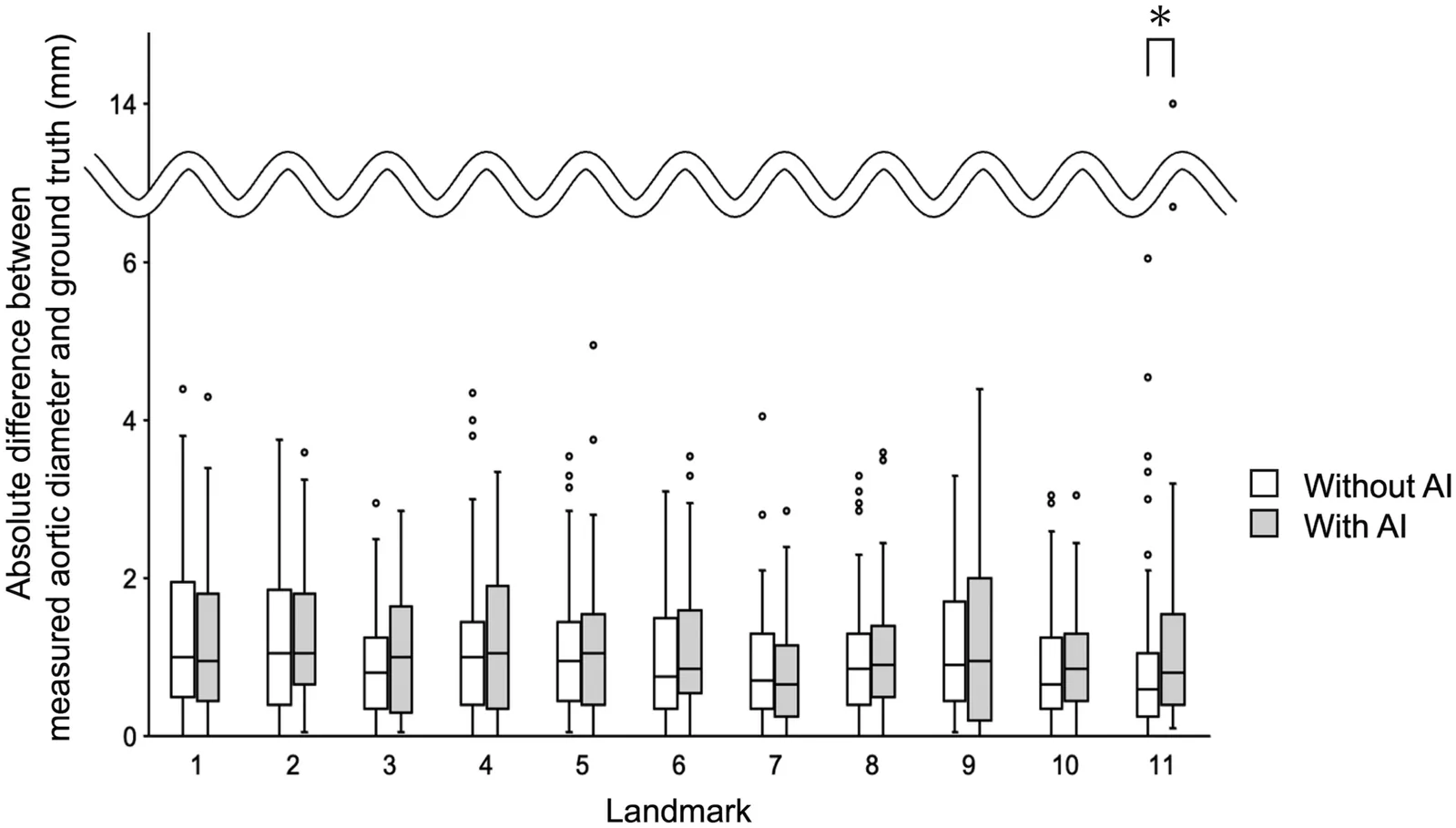
Comparison between absolute differences in aortic diameters between the methods. Comparison between the Diff_AI-assisted_ and the Diff_manual_ at each landmark. There was no evidence of a difference between Diff_AI-assisted_ and Diff_manual_ at landmarks 1 to 10, but Diff_AI-assisted_ was statistically larger than Diff_manual_ at landmark 11. The largest outlier of Diff_AI-assisted_ at landmark 11 (14 mm) was extremely larger than the other outliers. The boundaries of boxes closest to zero = 25th percentile, the line in boxes = median, the boundary of boxes farthest from zero = 75th percentile, error bars = smallest and largest values in 1.5 box lengths of 25th and 75th percentiles, small circles = outliers, and *P < .004. AI = artificial intelligence, Diff_AI-assisted_ = absolute difference in aortic diameter between the AI-assisted method on non-contrast computed tomography and the manual method on contrast-enhanced computed tomography, Diff_manual_ = absolute difference in aortic diameter between the manual method on non-contrast computed tomography and the manual method on contrast-enhanced computed tomography.

**Figure 4.**
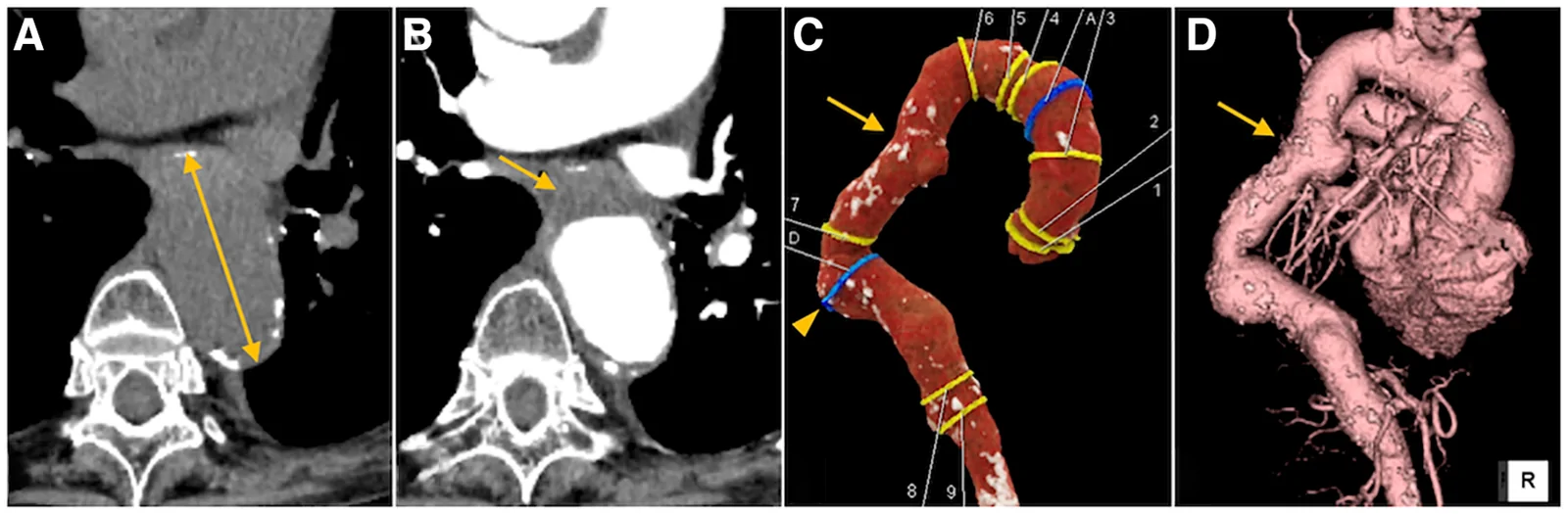
Thoracic aortic aneurysm in a 77-year-old female patient. An extremely large outlier of 14 mm was found in the absolute difference between the aortic diameter measured by the AI-assisted method on NCCT and ground truth at landmark 11. The actual section of the maximum diameter of the descending aorta on NCCT was shown. The length of the double-headed arrow is the correct maximum diameter (A). The same section on CECT showed a thick mural thrombus (arrow) (C). A 3-dimensional image of the aorta processed from NCCT by AI; the actual maximum diameter corresponding to the section in both (A) and (B) (arrow) was underestimated, and an incorrect location was denoted as the section with the maximum diameter of the descending aorta (arrowhead) (C). A 3-dimensional image of the aorta on CECT is shown for reference (B). AI = artificial intelligence, CECT = contrast-enhanced computed tomography, NCCT = non-contrast computed tomography.

The upper limits of the 95% CI of Diff_AI-assisted_ and Diff_manual_ for each of the 11 landmarks were less than the 1.5 mm cutoff. We found no evidence of a difference between Diff_AI-assisted_ and Diff_manual_ at landmarks 1 to 10. However, landmark 11 showed a difference between Diff_AI-assisted_ and Diff_manual_: Diff_AI-assisted_ (0.96 mm; 95% CI = 0.77–1.16) was larger than Diff_manual_ (0.71 mm; 95% CI = 0.55–0.89; *P* < .004; Table 2). Three relatively large outliers of approximately 5 mm or greater were observed, with Diff_AI-assisted_ of 4.95 mm at landmark 5 and 6.7 mm and 14.0 mm at landmark 11. The case with the largest Diff_AI-assisted_ (14 mm) was a patient with saccular dilatation and a thick mural thrombus in the descending aorta (Fig. 3). The AI showed an incorrect location as the cross-section with the maximum diameter and misled the 2 radiologists (Fig. 4).

**Table 2:** Mean aortic diameter at 11 landmarks in each measurement and comparison between Diff_AI-assisted_ and Diff_manual_.

Measurement	Aortic diameter (mm)*			Diff_AI-assisted_ (mm)‡	Diff_manual_ (mm)‡	*P* value
	AI-assisted	Manual	Ground truth†			Diff_AI-assisted_ versus Diff_manual_
Landmark						
1	34.4 ± 4.9	34.2 ± 4.7	34.2 ± 4.6	1.05 (0.87–1.24)	1.11 (0.92–1.11)	.61
2	31.7 ± 3.9	31.1 ± 4.0	31.1 ± 4.2	1.11 (0.94–1.29)	1.01 (0.82–1.21)	.34
3	37.1 ± 4.9	36.8 ± 5.1	36.3 ± 5.1	0.88 (0.73–1.05)	0.77 (0.64–0.91)	.21
4	36.3 ± 4.3	35.9 ± 4.6	35.4 ± 4.3	1.01 (0.82–1.22)	0.97 (0.80–1.15)	.68
5	32.5 ± 4.3	32.2 ± 4.3	31.7 ± 4.2	0.98 (0.81–1.17)	0.94 (0.79–1.11)	.67
6	30.9 ± 5.0	30.5 ± 5.1	30.2 ± 5.3	0.96 (0.80–1.12)	0.83 (0.68–0.98)	.15
7	28.1 ± 5.1	27.9 ± 5.2	27.7 ± 5.4	0.68 (0.56–0.81)	0.77 (0.63–0.93)	.27
8	26.0 ± 3.9	26.0 ± 4.0	25.7 ± 3.9	0.90 (0.76–1.06)	0.84 (0.70–0.99)	.53
9	24.7 ± 3.9	24.7 ± 3.6	24.4 ± 3.6	0.96 (0.76–1.19)	1.00 (0.84–1.17)	.76
10	38.0 ± 4.9	37.8 ± 5.3	37.5 ± 5.1	0.85 (0.72–0.99)	0.76 (0.62–0.90)	.20
11	32.8 ± 6.8	32.3 ± 7.0	32.3 ± 7.3	0.96 (0.77–1.16)	0.71 (0.55–0.89)	<.004

AI = artificial intelligence, CECT = contrast-enhanced computed tomography, Diff_AI-assisted_ = absolute difference in aortic diameter between the AI-assisted method on non-contrast computed tomography and the manual method on CECT, Diff_manual_ = absolute difference in aortic diameter between the manual method on non-contrast computed tomography and the manual method on CECT.

* Values are means ± standard deviation.

† Aortic diameter measured by the manual method on CECT.

‡ Values are geometric means with 95% confidence intervals in parentheses.

The intraclass correlation coefficients in the raw data of the measured aortic diameters between the 2 radiologists were manual, 0.95 (95% CI = 0.94–0.96); AI-assisted, 0.97 (95% CI = 0.97–0.98); and ground truth, 0.97 (95% CI: 0.96–0.97).

### 3.4. Evaluation time

**Figure 5.**
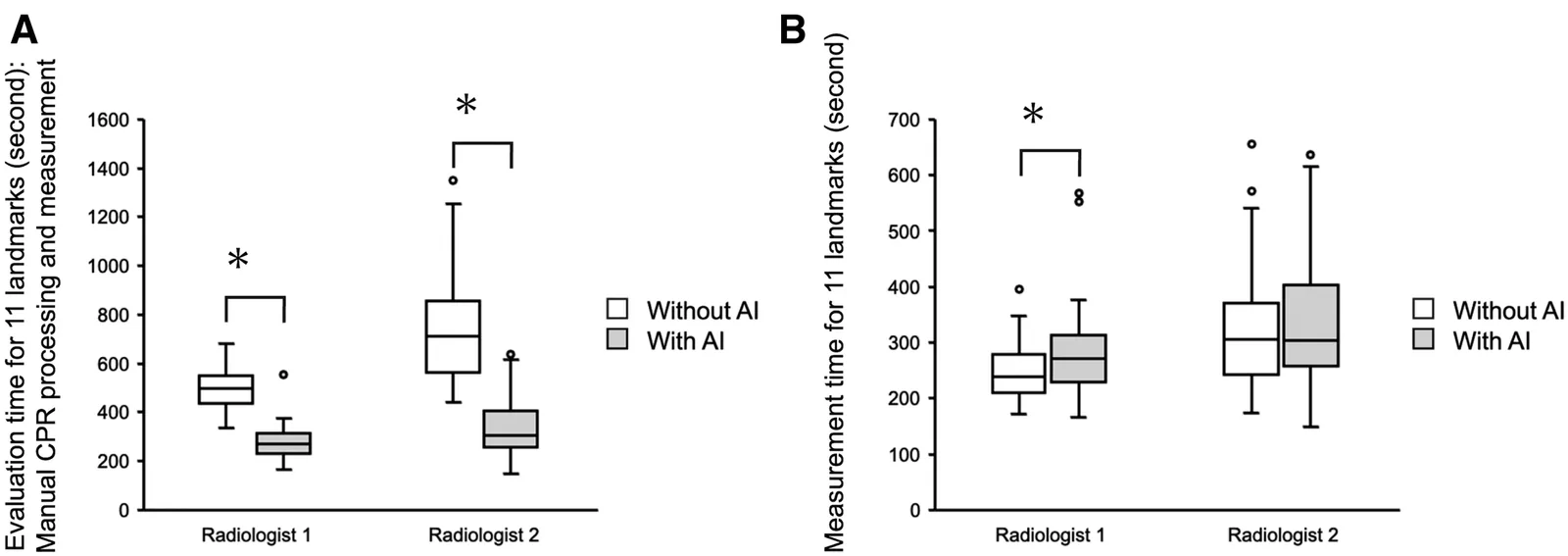
Evaluation and measurement times. Evaluation time included the entire duration of the process on NCCT, which was the sum of the times for manual CPR processing and the measurement itself (A). Measurement time included the duration of only the measurement process on NCCT (B). The boundary of boxes closest to zero = 25th percentile, the line in boxes = median, the boundary of boxes farthest from zero = 75th percentile, error bars = smallest and largest values in 1.5 box lengths of 25th and 75th percentiles, small circles = outliers, and *P < .001. AI = artificial intelligence, CPR = curved planar reformation, NCCT = non-contrast computed tomography.

The median evaluation times for the 11 landmarks among the 79 patients by the AI-assisted and manual methods on NCCT were 496 seconds (interquartile range [IQR] = 438–550) and 272 seconds (IQR = 229–313), respectively, by radiologist 1 (*P* < .001) and 710 seconds (IQR = 563–855) and 304 seconds (IQR = 257–404), respectively, by radiologist 2 (*P* < .001). The measurement times for determining aortic diameters using the manual method were 239 seconds (IQR = 210–279) for radiologist 1 and 305 seconds (IQR = 243–370) for radiologist 2. The measurement time for determining aortic diameter using the AI-assisted method was significantly longer than that using the manual method for radiologist 1 (*P* < .001). Conversely, no difference was found in the measurement time for aortic diameter between the AI-assisted method and manual method for radiologist 2 (*P* = .44; Fig. 5).

## 4. Discussion

Our study showed that AI-assisted aortic diameter measurement on NCCT was as accurate as manual measurement on CECT. In addition, the evaluation time using the AI-assisted method on NCCT was reduced compared with that using the manual method on NCCT. The upper limits of the 95% CI of Diff_AI-assisted_ were less than 1.5 mm regardless of the anatomical landmark. To our best knowledge, this is the first study to evaluate AI-assisted measurement – rather than that performed by only AI – which would be viable in clinical practice, and to compare AI-assisted measurement on NCCT with the “ground truth,” that is, the aortic diameter manually measured using CECT.

Although previous studies have evaluated the performance of aortic diameter measurement using AI, these studies focused on AI-only measurement and did not include AI-assisted measurement. The coefficients of repeatability in these studies were large and varied. This may be because the results of AI-only measurements were adopted as they were, particularly for landmarks 1 and 2.^[8,15,16]^ In fact, for landmarks 1 and 2, which are anatomically highly tortuous and susceptible to heartbeats, more than 50% of the AI-only measurements required correction by the readers.^[12]^ Diff_AI-assisted_ values at landmarks 1 and 2 were relatively larger than those at other landmarks, and AI-assisted measurement improved the accuracy and decreased variation, particularly at these complex landmarks and in challenging cases, even when using NCCT.

Conversely, AI does not come without disadvantages. Although Diff_AI-assisted_ was less than 1.5 mm at each landmark, Diff_AI-assisted_ was statistically larger than Diff_manual_ at landmark 11. The largest outlier of Diff_AI-assisted_ (14.0 mm) at landmark 11 in 1 patient involved 2 localized dilatations in the descending aorta: 1 fusiform and 1 saccular. Although the actual cross-section with the largest diameter was the section with the saccular aneurysm, the diameter was underestimated by AI owing to the thick mural thrombus. This may be because landmark 11 is not anatomically defined; the AI generates orthogonal reformats perpendicular to the aortic centerline based on the aortic segmentation, and the aortic diameter is determined on these reformats. The descending aorta has the longest distance of cross-sections to explore, including the maximum diameter, making such errors more likely to occur. In the other 2 cases with relatively large outliers, thick mural thrombus was also present at the corresponding sites. These findings suggest that the interpretation of AI-assisted measurements may require caution in patients with thick mural thrombus. Overall, most of the observed measurement differences were small and unlikely to be clinically meaningful. However, even small discrepancies could potentially influence management decisions in patients whose aortic diameter is close to a treatment threshold. Therefore, AI-assisted measurements should be interpreted with caution in such borderline cases.

In addition, the root of the aorta could not be recognized by AI in 1 patient who was excluded from this study owing to a lack of analysis. The patient had pleural effusion and a large esophageal hiatal hernia. The factors influencing the AI analysis were found not only in the aorta but also in the surrounding region.

AI showed the cross-sections perpendicular to the centerline of the aorta at the 11 landmarks and the maximum aortic diameters corresponding to the cross-sections. The evaluation time for AI was reduced by eliminating the time required for manual CPR processing and searching for anatomical locations. However, AI-assisted measurement times tended to be longer. We propose 2 reasons for this phenomenon. First, AI results were not directly adopted as the measured value. Rather, all AI-assisted measurements were performed manually, using the AI outputs only as a reference. Second, the presented cross-section was not always correctly located at the corresponding landmark. Therefore, correcting the AI measurements required additional time.

This study has several limitations. First, this was a single-center study, and only 1 CT vendor was included. Multicenter studies with larger sample sizes are required to validate our findings. In addition, although descriptive subgroup analyses according to disease category were performed, the limited sample size within each subgroup precluded robust statistical comparisons. Second, all aortic diameters were measured using non-ECG-gated NCCT and CECT because patients in this study were not clinically indicated for ECG-gated scans. The measurements, particularly at landmarks 1 and 2, might not be entirely accurate due to motion artifacts. Third, the evaluation time by the AI-assisted method did not include the time required for AI analysis of the original image. The analysis was completed within approximately 10 minutes of scanning; no obvious problems occurred with the actual reading. Fourth, our analysis did not include evaluation using the largest short-axis diameter, which is commonly used in clinical practice but is not guideline-recommended, because the AI-assisted method used in this study measures the maximum diameter on planes perpendicular to the aortic centerline. Future studies examining this topic are required to further explore this type of analysis and validate our findings.

In conclusion, AI-assisted thoracic aortic diameter measurement on NCCT was as accurate as manual measurement on CECT; the evaluation time using the AI-assisted method on NCCT was shorter than that of the manual method on NCCT. Using AI assistance, aortic diameter measurement could be performed effectively on NCCT, making this imaging strategy useful for screening and follow-up examinations and managing patients at potential risk of adverse reactions to contrast media.

## Author contributions

**Conceptualization:** Akiyoshi Suzuki, Nobuo Tomizawa.

**Data curation:** Akiyoshi Suzuki, Nobuo Tomizawa, Kotaro Yamamoto, Yuichi Morita, Shohei Fujita.

**Formal analysis:** Akiyoshi Suzuki, Nobuo Tomizawa.

**Funding acquisition:** Kanako K. Kumamaru.

**Investigation:** Akiyoshi Suzuki, Nobuo Tomizawa.

**Methodology:** Akiyoshi Suzuki, Nobuo Tomizawa.

**Project administration:** Akiyoshi Suzuki, Nobuo Tomizawa, Shigeki Aoki.

**Resources:** Keiichi Funaki, Jonathan Sperl.

**Software:** Keiichi Funaki, Jonathan Sperl.

**Supervision:** Nobuo Tomizawa, Katsuhiro Sano, Toshiaki Akashi, Mitsuo Nishizawa, Kazuhiro Suzuki, Akihiko Wada, Daisuke Endo, Satoshi Matsushita, Kanako K. Kumamaru, Minoru Tabata, Shigeki Aoki.

**Validation:** Akiyoshi Suzuki.

**Writing** – **original draft:** Akiyoshi Suzuki.

**Writing** – **review & editing:** Akiyoshi Suzuki, Nobuo Tomizawa.
